# Life-Course Approach to Vaccination in Bangladesh for Meeting the Health and Health-Related Sustainable Development Goals: A Commentary

**DOI:** 10.1093/infdis/jiab455

**Published:** 2021-09-22

**Authors:** Taufiqur Rahman Bhuiyan, Taufiqul Islam, Firdausi Qadri

**Affiliations:** 1 International Centre for Diarrhoeal Disease Research, Bangladesh, Dhaka, Bangladesh; 2 School of Medical Science, Griffith University, Gold Coast, Australia

**Keywords:** cholera, vaccine, emerging diseases, Bangladesh

## Abstract

Bangladesh is entering from low-income to lower-middle-income status in 2020, and this will be completed in the next 5 years. With gross national income growing, vaccines will need to be procured through private market for the Expanded Program on Immunization. A cost-benefit analysis is needed to evaluate vaccine demand in different socioeconomic groups in the country, to inform this procurement. Moreover, disease burden studies and awareness of importance of specific vaccines are needed as we move forward. A life-course approach to vaccination may enable whole society to realize the full potential of vaccination and address most significant threats to its success over time.

Immunization and vaccination offer people of all ages of life a range of benefits depending on their stage in life and specific disease risks. By using a life-course approach, immunizations can be better incorporated into the primary health care system of Bangladesh and make a powerful contribution to the success of achieving the sustainable development goals (SDGs). Taking a life-course approach to immunization offers an important opportunity to improve the healthcare system and promote economic sustainability. In addition, vaccines play a vital complementary role for global public health success, including the eradication of smallpox and reductions of infection due to polio, diphtheria, tetanus, pertussis, and measles [1]. No other health intervention has the potential to reach as many people globally as immunization. Improving health is an ongoing process and at each stage, some interventions have a bigger impact than others. Despite the benefits of a life-course approach to vaccinations as per the recommendations of the Global Vaccine Action Plan 2011–2020, many countries have not yet implemented this approach. Despite some progress in this area, there is an urgent need to readdress the situation over the next decade of 2021–2030, and the vision “Leave No One Behind” in immunization is important for all countries including Bangladesh. Therefore, to encourage sustainable policies and programs, implementation of a life-course approach to immunization should be undertaken by policymakers, partners, funding agencies, and other stakeholders.

## SUCCESS OF EXPANDED PROGRAM ON IMMUNIZATION ACHIEVED IN BANGLADESH

Bangladesh has achieved success in meeting most targets of the immunization strategies related to the Millennium Development Goals and is on track for most of the indicators. However, immunization has so far been mainly focused on infants and young children. To meet the SDGs targeted for 2030, immunization policy now needs to be planned for individuals of all ages, examining the specific benefits achieved at different stages in life and any specific risks. A life-course vaccination approach to immunization should be extended beyond infancy to all ages and incorporated into the primary health care system, to help build a foundation for universal health coverage and contribute powerfully to the success of the SDGs. A life-course vaccination approach to immunization will improve healthcare delivery and promote economic growth and prosperity. Such a life-course approach requires that immunization schedules and access to vaccination need to be based on an individual’s phase in life, lifestyle, and specific risks for infectious diseases that participants may encounter. Bangladesh has announced a comprehensive immunization plan that follows this life-course approach, which is shown in Table 1.

**Table 1.** Comprehensive Multiyear Immunization Plan for Bangladesh, 2018–2022

Immunization Plan	Status
1. Strategic plan for elimination of measles, rubella and congenital rubella syndrome by 2023	Developed
2. National immunization policy	Developed
3. New introduction of vaccines (HepB, Hib, pentavalent^a^, MCV2, PCV, IPV, fIPV, HPV Demo.)	Introduced in routine immunization from 2003 to 2016
4. National Immunization Technical Advisory Group (15 members)	Functional
5. National Verification Committee for Measles and Rubella and NCCPE for Polio	Functional
6. Spending on vaccines financed by the government	27%
7. Spending on routine immunization program financed by the government	29%
8. Updated microplans that include activities to improve immunization coverage	64 districts (100%)
9. National policy for health care waste management including waste from immunization	In place
10. Most recent EPI coverage evaluation survey	Published February 2020

Abbreviations: EPI, Expanded Program on Immunization; fIPV, fractional dose of inactivated polio vaccine; HepB, hepatitis B vaccine; Hib, *Haemophilus influenzae* type b vaccine; HPV Demo., Human Papillomavirus Vaccine Demo; IPV, inactivated polio vaccine; MCV2, Measles-Containing-Vaccine second-dose; NCCPE, National Certification Committee for Polio Eradication; PCV, pneumococcal conjugate vaccine.

^a^Pentavalent vaccine: diphtheria-tetanus-pertussis, HepB, and Hib.

## LIFE-COURSE APPROACH TO VACCINATION MAKES SENSE

Vaccines are the best protection against infection, especially those that lead to serious complications (including malignancies) and death [2]. The United States (US) Centers for Disease Control and Prevention recommends vaccinations to protect children against 14 infectious diseases (measles, mumps, rubella, chickenpox, hepatitis A, hepatitis B [HepB], diphtheria, tetanus, pertussis, *Haemophilus influenzae* type b [Hib], polio, influenza, rotavirus, and pneumococcal disease) before the age of 2 years. Combination vaccines, where 2 or more different vaccines are combined into a single shot, has been found to have both financial and programmatically beneficial effects. In the US, combination vaccines have been used since the mid-1940s. Examples of such combination vaccines include diphtheria-tetanus-pertussis (DTap), trivalent inactivated polio vaccine (IPV), measles-mumps-rubella, DTap-Hib, and Hib-HepB. In Bangladesh, the Expanded Program on Immunization (EPI) was launched on 7 April 1979, and has been intensified both in rural and urban areas in phases since 1985. Tetanus vaccination was introduced for women of reproductive age in 1993. Polio eradication and maternal and neonatal tetanus elimination (MNTE) activities started in 1995. Gavi introduced autodisable/single-use syringes in 2004; HepB vaccination was introduced in selected districts in 2004 and nationwide by 2005; pentavalent (DTap-HepB-Hib) vaccination was introduced in 2009; measles-rubella vaccine and measles vaccine, second dose (MSD) were introduced in 2012; pneumococcal conjugate vaccine and IPV were introduced in 2015; and a measles-rubella catch-up campaign was held in 2014. In 2015, the EPI program switched from MSD to MCV2 (measles second dose), and switched from trivalent oral polio vaccine (OPV) to bivalent OPV in 2016, and finally to fractional dose of IPV in November 2017. In 2016, Bangladesh carried out a demonstration project on the human papillomavirus (HPV) vaccine in school-aged girls in Gazipur. Bangladesh received MNTE certification from the World Health Organization (WHO) in 2008.

It has been shown that no chronic health problems have been observed after receiving several vaccines together [3]. In addition, a number of studies have reported safety data of multiple combinations of vaccines that were licensed in children of different ages [2, 3]. Recommended vaccines have been shown as effective in combination as they are given individually [4, 5]. It should be noted that some combination vaccines may cause some adverse effects such as fever or occasionally febrile seizures, but these have not caused any long-term harm to participants. Based on this evidence, the Advisory Committee on Immunization Practices of the WHO and the American Academy of Pediatrics recommend administering all routine childhood vaccines on time. Additional areas of focus for improving vaccination programs include robust data capture, broader involvement of healthcare professionals, public perceptions of vaccination, and more robust vaccine introduction into school and workplace settings [6].

In 2005, 72 of the world’s poorest nations spent US$2.5 billion on vaccination, up from US$1.1 billion in 2000. Annual vaccination expenditures was expected to rise to around US$4.0 billion by 2015. The total cost of vaccination from 2006 to 2015 was projected to be US$35 billion. Total revenue for 2006–2015 in all 117 low- to middle-income countries (LMICs) was anticipated to be US$76 billion, including US$49 billion for sustaining present systems and $27 billion for efficiency. Rotavirus hospitalizations and fatalities were remarkably reduced in 2016 in all African nations by implementing rotavirus vaccines [7]. In 2003, it has been shown that vaccinations saved approximately tens of billions of dollars in revenues throughout the world [8]. Indirect costs were also reduced, including lost productivity, reduced antibiotic use, multidrug-resistant strains, and the development of new vaccines against infectious diseases from pathogens that are resistant to antibiotics [7, 9–11]. A robust immunization program is essential for robust public health and will help to alleviate inequity and poverty.

## IMPORTANT GOALS FOR BANGLADESH TO ELIMINATE INFECTIOUS DISEASES BY 2030

In Bangladesh as well as in other LMICs, vaccination at different stages of the life-course needs to be implemented. This cycle starts with vaccination of pregnant women with tetanus, diphtheria, and pertussis vaccines (Td, DTaP) as well as seasonal influenza. Vaccination of newborns with HepB and OPV can decrease transmission of infections from mothers to infants. Vaccination around 9 months of age against typhoid fever, meningococcal disease, rabies, yellow fever, and seasonal influenza are options that can be introduced based on susceptibility to these infections in particular regions within the country or on a national basis to decrease the burden of these diseases. In the second year of life (12–23 months), the first booster of diphtheria, pertussis (whooping cough), and tetanus (DPT), as well as vaccinations against cholera, hepatitis A, mumps, varicella, meningococcal disease, rabies, typhoid fever, and seasonal influenza are options. Children (aged 2–5 years) benefit from the second booster dose of a tetanus vaccine (Td), as well as the 23-valent pneumococcal vaccine, and vaccines against cholera, rabies, typhoid fever, and seasonal influenza. Adolescents aged 5–19 years benefit from vaccination against cholera, HPV, rabies, typhoid fever, seasonal influenza, and dengue as well as a third booster dose with Td. Adults benefit from vaccination against cholera, rabies, typhoid fever, seasonal influenza, and dengue to protect against endemic infections. This is the time to rethink to develop unified vaccination policies for both children and older adults. The burden of adult disease is often exhibited based on the experience of high-income countries. Currently, use of seasonal influenza vaccines in adults aged >65 years in high-income countries ranges from <3% to >80% and is even more limited in LMICs [12]. This may be due to lack of awareness and having less information on the benefits of immunization for older adults. Therefore, a holistic perspective on the needs of implementation of vaccination is needed for older adults, as the United Nations Department of Economic and Social Affairs reported that by 2050, the world’s adult population (>65 years) will double [13].

## VACCINES THAT NEED FURTHER DEVELOPMENT OR DISCUSSION FOR INCLUSION IN THE NATIONAL IMMUNIZATION PROGRAM

Vaccines in the pipeline such as those against respiratory syncytial virus, Nipah virus, and Japanese encephalitis virus may protect against death and morbidity. Discussions have already started at the National Immunization Technical Advisory Group for evaluating the need of typhoid conjugate vaccine and its introduction in Bangladesh. Both catch-up campaigns (9 months–15 years) and routine immunization will be needed in Bangladesh. Typhoid vaccine availability for the targeted children in Bangladesh may be an issue, until new vaccines become prequalified for use. Use of the rotavirus vaccine is also important, as studies show that the vaccine is effective and can reduce out-of-pocket expenditures that are estimated to be very high in Bangladesh [14].

## INTRODUCTION OF CHOLERA VACCINE IN BANGLADESH

An initiative undertaken by the Global Task Force on Cholera Control proposes to reduce cholera deaths by 90% and eliminate cholera in at least from 20 countries by 2030 [15]. A Global Roadmap to 2030 has been outlined for prevention and control of cholera [16]. We have reported that a single dose of the inactivated whole-cell oral cholera vaccine (OCV) was efficacious for at least 2 years in children 5 years or older and in adults in a setting with a high level of cholera endemicity [17, 18]. Younger children may have a lower frequency of preexisting natural immunity to explain the lessened efficacy in these young children. Cholera vaccine strategies will be targeted to all age groups, although high-risk groups such as the elderly, exposed populations, and healthcare workers may need to be targeted to meet the SDG to “Leave No One Behind” from vaccination. OCVs will play a vital role to control cholera, although long-term progress in water, sanitation, and hygiene services and behavior change should be the eventual objectives [19].

## Conclusions

Different countries are at different levels of implementation for life-course vaccination programs, and innovative approaches are being implemented that may inform immunization policies. Receiving vaccines at the recommended time of life will maximize protection for the individual as well as protecting family members and friends, particularly those with lessened immune systems such as the elderly and newborns. In this approach, the availability of safe and affordable vaccines as well as local production are important considerations.

**Figure 1.**
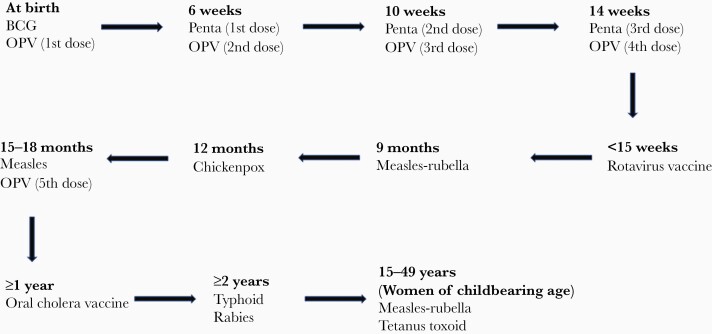
Life-course approach for vaccine recommendation in Bangladesh. List of vaccines administered in Bangladeshi population from birth throughout the life. Abbreviations: OPV, oral polio vaccine; Penta, pentavalent vaccine (diphtheria-tetanus-pertussis, hepatitis B, and *Haemophilus influenzae* type b).

Such a life-course approach to vaccination needs to be incorporated into the national plan of the Ministry of Health and Family Welfare with the government (Figure 1). The coronavirus disease 2019 (COVID-19) pandemic has made the life-course approach to vaccination even more important. Access to COVID-19 vaccines in LMICs is one of the most important market failures; therefore, public-private partnerships, as well as industry incentives, need to be stronger to develop and deliver global public goods. Policy makers have emphasized the importance of increasing allocations for vaccines, family planning, and strengthening governance of health systems by recruiting a skilled workforce considering Universal Health Coverage Vision 2041. After the start of the COVID-19 pandemic, most if not all demonstration vaccination campaigns/programs have been halted. As COVID-19 vaccination has started on a large scale in Bangladesh, reactivation of earlier demonstration vaccination projects, including coadministration vaccine approaches with pneumococcal or influenza vaccines and tetanus vaccine (for pregnant women), need to be pursued since we do not have any information about these strategies. Different sponsors, collaborators, and stakeholders have already started discussion for initiating the process and securing funds for other vaccination programs along with COVID-19 vaccination. A faster and more effective process is needed to end the acute phase of the COVID-19 pandemic [8].

## Notes

***Acknowledgments.*** The International Centre for Diarrhoeal Disease Research, Bangladesh (icddr,b) is grateful to the National Institutes of Health (NIH) for its support toward its research efforts. icddr,b is also grateful to the governments of Bangladesh, Canada, Sweden, and the United Kingdom for providing core/unrestricted support.

***Financial support.*** This research was supported by the NIH (Global Emerging Leader Award number K43TW010362 to T. R. B. and grant number R01 AI130378 to T. R. B.) and the icddr,b. This publication was also supported by the Bill & Melinda Gates Foundation (INV-033599).

***Potential conflicts of interest.*** All authors: No reported conflicts.
